# Obstetric brachial plexus injuries (OBPIs): health-related quality of life in affected adults and parents

**DOI:** 10.1186/s12955-018-1039-z

**Published:** 2018-11-15

**Authors:** Christopher W. H. Yau, Elena Pizzo, Chetankumar Prajapati, Tim Draycott, Erik Lenguerrand

**Affiliations:** 1 The Chilterns, Southmead Hospital, Bristol, BS10 5NB UK; 2 Translational Health Sciences, University of Bristol, Bristol, BS2 8DZ UK; 3 Department of Applied Health Research, University College London, London, WC1E 7HB UK; 4 Bristol Medical School, University of Bristol, Bristol, BS8 1TH UK

**Keywords:** Obstetric brachial plexus injury, Quality of life, Obstetrics

## Abstract

**Background:**

Obstetric brachial plexus injuries (OBPIs) are rare but can have significant implications for those affected, their caregivers and the health system. Symptoms can range from restricted movement to complete paralysis of the arm. We investigated health-related quality of life in adults with OBPIs and parents of children with permanent OBPIs, compared these with population norms, and investigated whether certain socio-demographic or clinical factors were associated with the quality of life in these cohorts.

**Methods:**

A cross-sectional study examined 50 affected adults and 78 parents. Participants completed EQ-5D-5 L and characteristics questionnaires. EQ-5D-5 L responses were mapped onto an EQ-5D-3 L value set to generate utility scores. Mean utility scores were compared with English population norms. Univariable and multivariable linear regression models were conducted to assess for associations between participant characteristics and the utility scores.

**Results:**

The overall mean utility scores for affected adults and parents were 0.56 (SD 0.28) and 0.80 (SD 0.19) respectively. Affected adults (95% CI (− 0.38, − 0.22), *p* < 0.001) and parents of children with permanent OBPIs (95% CI (− 0.10, − 0.02), *p* = 0.007) had lower mean utility scores, and therefore quality of life, compared to English population norms. For affected adults, previous OBPI surgery (95% CI (0.01, 0.25), *p* = 0.040), employment in non-manual work (95% CI (0.06, 0.30), *p* = 0.005) and having a partner (95% CI (0.04, 0.25), *p* = 0.009) appeared to be positively associated with the utility score. Affected adults receiving disability benefits related to OBPIs appeared to have worse utility scores than those not receiving any disability benefits (95% CI (− 0.31, − 0.06), *p* = 0.005). For parents, employment was associated with better utility scores (95% CI (0.02, 0.20), *p* = 0.024) but the presence of one or more medical condition appeared to be associated with worse utility scores (95% CI (− 0.16, − 0.04), *p* = 0.001).

**Conclusions:**

Adults with OBPIs and parents of children with permanent OBPIs reported worse utility scores, and therefore quality of life, compared to the English general population. We also identified certain characteristics as possible factors to consider when dealing with utility scores in these cohorts. The utility scores in this study can be used in future economic evaluations related to OBPIs.

**Electronic supplementary material:**

The online version of this article (10.1186/s12955-018-1039-z) contains supplementary material, which is available to authorized users.

## Background

Obstetric brachial plexus injuries (OBPIs) are rare [1, 2] but can have significant implications for those affected [3], their caregivers [4] and the wider health system [5]. These injuries – sometimes referred to as Erb’s palsies – can occur as a result of the mismanagement of an obstetric emergency: shoulder dystocia; where the baby’s head is delivered but its anterior shoulder is impacted against the mother’s pelvic bone [6]. Inappropriate management of this emergency can lead to damage to the brachial plexus, a collection of nerves on either side of the neck which are responsible for sensation and movement of the arm [7]. Symptoms can range from restricted movement in the shoulder, elbow and wrist, to complete paralysis of the arm [1, 3]. They can also be transient or permanent (lasting for longer than 12 months after birth) [1, 8]. In England (2000 to 2010), litigation costs associated with shoulder dystocia and OBPIs amounted to over £103 million [5]. This is money that could otherwise have been spent on frontline patient care.

These potentially life-changing injuries warrant investigation into how they affect the quality of life of both sufferers and their caregivers using formal quality of life utility scores. In this context, utility is used to describe the preferences of individuals for a particular set of health outcomes and is way of valuing different health characteristics on a single scale [9, 10]. To date, the only quality of life utility scores related to OBPIs have been assigned at a distance, by healthcare professionals from outside the specialties involved in OBPIs [11]. There has been no direct assessment of the impact of OBPIs on individuals and although there is a growing body of research investigating the effects of OBPIs on families and caregivers [4, 12, 13], it has never been expressed using utility scores.

We used a generic, preference-based health status instrument (EQ-5D-5 L) to determine the quality of life utility scores of affected adults and parents of children with permanent OBPIs. The utility scores of these cohorts were compared with population norms. We also investigated whether certain socio-demographic or clinical factors were associated with the utility value.

## Methods

### Participants

Affected adults (≥18 years old) and parents of children with permanent OBPIs (lasting for 12 months or more after birth) were recruited through, and in collaboration with, the Erb’s Palsy Group (UK charity) [14]. The Erb’s Palsy Group recruited participants and advertised the study on their website and social media pages. Participants were also recruited at an annual Erb’s Palsy Group event.

All participants were asked to complete an EQ-5D-5 L questionnaire [15] as well as a characteristics questionnaire by telephone or post. The EQ-5D -5 L questionnaire is a generic, preference-based instrument assessing health over five dimensions: mobility, self-care, usual activities, pain/discomfort and anxiety/depression. Within each health dimension, there are five levels of functionality: no problems, slight problems, moderate problems, severe problems or extreme problems/unable to function [15]. The 5 L version was chosen over the 3 L version (with only 3 levels for each health dimension) as it has been developed to try and improve the instrument’s sensitivity in detecting small changes in health [15]. The characteristics questionnaire included socio-demographic information and clinical details. The characteristics questionnaire for the parents also included questions regarding the child that was being cared for. A non-medical administrator, separate from the Erb’s Palsy Group, contacted participants who wished to complete the questionnaires by telephone.

A total of 50 affected adults were initially recruited for this study. Two participants were excluded as they had incomplete EQ-5D-5 L scores, leaving a total of 48 affected adults for calculating the utility scores. A further 6 adults (13%) were then excluded as their questionnaires contained missing data, leaving 42 participants for the subsequent analyses. All of the affected adult characteristics are summarised in Table 1.

**Table 1:** Affected adult characteristics

Characteristics	Total (*n* = 50)
Affected adults	n (%)
Age (years)^a^	28 (24, 46)
Age subcategories (years)	
≤ 24	18 (36)
25–39	14 (28)
40–54	6 (12)
≥ 55	12 (24)
Gender	
Male	14 (28)
Female	36 (72)
Marital status	
No partner	33 (66)
With partner	17 (34)
Education status	
Secondary school/ college	20 (40)
Higher education	29 (58)
Missing data	1 (2)
Employment status	
Manual work	6 (12)
Non-manual work	18 (36)
Not working	22 (44)
Missing data	4 (8)
Disability benefits	
Receives benefits related to OBPIs	19 (38)
Receives non-related benefits	1 (2)
Receives no benefits	29 (58)
Missing data	1 (2)
Handedness	
Left	19 (38)
Right	31 (62)
Injury site	
Left	26 (52)
Right	23 (46)
Both left and right	1 (2)
Previous OBPI surgery	
Yes	32 (64)
No	18 (36)
Narakas (severity score)	
Know Narakas score	12 (24)
Don’t know Narakas score	36 (72)
Missing data	2 (4)
Has ≥1 medical condition	33 (66)

^a^Median, 25th, and 75th centiles presented as the distribution for age was skewed

Overall, 78 parents were initially recruited for this study. One participant with an incomplete EQ-5D-5 L score was excluded, leaving 77 parents for calculating the utility scores. A further 8 parents (10%) were then excluded as their questionnaires contained missing data, leaving 69 parents for the remaining analyses. The characteristics for the parents and the children they looked after are summarised in Tables 2 and 3.

**Table 2:** Parent characteristics

Characteristics	Total (*n* = 78)
Parent	n (%)
Age (years)^a^	40 (8)
Age subcategories (years)	
≤ 35	24 (31)
36–45	35 (45)
≥ 46	19 (24)
Gender	
Male	6 (8)
Female	72 (92)
Relationship to child	
Father	6 (8)
Mother	72 (92)
Marital status	
No partner	19 (24)
With partner	58 (74)
Missing	1 (1)
Education status	
Secondary school/ college	41 (53)
Higher education	33 (42)
Missing	4 (5)
Employment status	
Working	64 (82)
Not working	13 (17)
Missing	1 (1)
Has ≥1 medical condition	45 (58)

^a^Mean and standard deviation presented as parent age had a normal distribution

**Table 3:** Child characteristics

Characteristics	Total (n = 78)
Child	n (%)
Age (years)^a^	8 (2,13)
Age subcategories (years)	
1	10 (13)
2–5	25 (32)
6–12	22 (28)
13–17	21 (27)
Gender	
Male	35 (45)
Female	43 (55)
Education status	
Too young for school	28 (36)
Primary school	19 (24)
Secondary school/college	30 (38)
Missing	1 (1)
Narakas (severity score)	
Know Narakas score	30 (38)
Don’t know Narakas score	46 (59)
Missing	2 (3)
Previous OBPI surgery	
Yes	55 (71)
No	23 (29)
Has ≥1 medical condition	
Yes	33 (42)
No	44 (56)
Missing	1 (1)

^a^Median, 25th, and 75th centiles presented as the distribution for age was skewed

### Utility scores

The responses from the EQ-5D-5 L questionnaires were mapped onto an EQ-5D-3 L value set [16] to generate utility scores. This has been recommended by the National Institute for Health and Care Excellence (NICE) in England [17, 18]. This mapping was performed using the *eq5dmap* command in Stata [19]. Mean utility scores for affected adults and parents of children with OBPIs were compared with population norms from the 2008 Health Survey for England [20] using Welch’s two-sample t-test.

### Associations between utility score and participant characteristics

The investigations were conducted separately for affected adults and parents, using the same strategy, which is described below. Univariable and multivariable linear regressions were conducted to assess for associations between participant characteristics and the utility scores. Robust standard errors (using Huber-White sandwich estimator) were used for all the regressions. The strength of evidence against the null hypothesis (that there is no association between the variable and the utility score) was indexed by the *p*-value derived from the regressions, with increasing evidence against the null hypothesis with decreasing *p*-values [21]. Variables with weak to strong evidence of association (p-value ≤0.10) with the utility score in the univariable analyses were considered for inclusion in the initial multivariable linear regression models. The following characteristics were also included in the initial multivariable regression model, regardless of their strength of association in the univariable analyses:

- Age (for affected adults and parents)
- Gender (for affected adults and parents)
- Marital status (for affected adults and parents)
- Employment status (for affected adults and parents)
- Education status (for affected adults and parents)
- Presence of one or more medical condition (for affected adults and parents)
- Child age (for parents)
- Presence of one or more child medical condition (for parents)

Age, gender, marital status, employment status and education status have previously been demonstrated to influence perceptions of health [22–24]. The presence of medical comorbidities was also deemed to be an important characteristic to include in the initial multivariable models.

Previous OBPI surgery was chosen as a variable of interest because it is a proxy for the severity of the condition; more severe injuries are more likely to require surgery. Previous OBPI surgery was forced into the multivariable models for the affected adults and the parents. Originally, the Narakas score (a classification system for OBPIs) [3] was selected, a priori, as an important characteristic for investigation. However, a high proportion of participants did not know their Narakas score (72% adults and 59% parents), and therefore it was not possible to perform any meaningful analyses with this characteristic.

The influence of each characteristic on the initial multivariable regression models was investigated. Variables were retained in the final multivariable models if their *p*-value was ≤0.05 (Wald test) and/or if their removal impacted the effect size of the other parameters by > 30%. Influential observations in the final models were identified using Cook’s distance and removed. In the final multivariable model, *p*-values around 0.05 were assumed to represent weak evidence against the null hypothesis and p-values < 0.001 as strong evidence [21].

Stata version 14.2 (StataCorp, Texas, USA) and Microsoft® Excel for Mac were used to perform the analyses. The University of Bristol Faculty of Health Sciences Research Ethics Committee approved this study (39641 and 39681). We obtained informed consent (written or e-mail) from all participants.

## Results

### Utility score (Table 4)

The overall mean utility score for affected adults was 0.56 (SD 0.28). The mean utility scores for males and females were 0.64 (SD 0.21) and 0.53 (SD 0.30) respectively. When compared with the general population, affected adults had worse utility scores (and therefore quality of life) (95% CI (− 0.38, − 0.22), *p* < 0.001). This remained the case when comparing by male (95% CI (− 0.37, − 0.11), *p* = 0.001) and female (95%CI (− 0.42, − 0.22), p < 0.001) subcategories.

**Table 4:** Mean utility scores

	General population norm^a^		Affected adult utility score (*n* = 48)					Parent utility score (*n* = 77)				
	Mean	SD	Mean	SD	Difference	95% CI	*p*-value	Mean	SD	Difference	95% CI	*p*-value
Overall	0.86	0.24	0.56	0.28	−0.30	−0.38, −0.22	< 0.001	0.80	0.19	−0.06	−0.10, − 0.02	0.007
Male	0.88	0.16	0.64	0.21	−0.24	− 0.37, − 0.11	0.001	0.89	0.15	0.01	−0.15, 0.17	0.877
Female	0.85	0.18	0.53	0.30	−0.32	− 0.42, − 0.22	< 0.001	0.79	0.19	− 0.06	− 0.11, − 0.01	0.010

^a^Obtained and calculated from 2008 Health Survey for England [18]

The overall mean utility score for parents of children with permanent OBPIs was 0.80 (SD 0.19), which was lower than the population norm (95% CI (− 0.10, − 0.02), *p* = 0.007), also suggesting poorer quality of life compared to the general population. The mean utility score for fathers was 0.89 (SD 0.15) but there was no evidence of a difference compared to the general male population (95% CI (− 0.15, 0.17), *p* = 0.877). For mothers, the utility was 0.79 (SD 0.19) suggesting that mothers had worse utility scores (and therefore quality of life) compared to the general female population (95% CI (− 0.11, − 0.01), *p* = 0.010).

### Associations between the utility score and participant characteristics (Table 5)

Previous OBPI surgery was not associated with the affected adult utility score in the univariable regression model (95% CI (− 0.21, 0.09), *p* = 0.411). Details of the affected adult univariable regression analyses can be found in Additional file 1. Employment status, marital status, previous OBPI surgery and disability benefits status were all included in the final multivariable model for adults with OBPIs. The backward elimination steps used to determine this final model are presented in Additional file 2. Three influential observations were removed from the final model. There was some evidence that adults with previous OBPI surgery had better utility scores than those who had no surgery after adjusting for the other variables in the final model (95% CI (0.01, 0.25), *p* = 0.040). Affected adults in non-manual jobs also appeared to have better utility scores than those without employment after adjusting for previous OBPI surgery, marital status and disability benefits status (95% CI (0.06, 0.30), *p* = 0.005). There was no evidence of any difference in utility scores between affected adults in manual work and those without employment (95% CI (− 0.14, 0.17), *p* = 0.835). Affected adults with a partner also had better utility scores than those without partners after accounting for the other variables in the model (95% CI (0.04, 0.25), *p* = 0.009). In contrast, affected adults receiving disability benefits related to their injury had worse utility scores and therefore quality of life compared to those receiving no disability benefits (95% CI (− 0.31, − 0.06), p = 0.005).

**Table 5:** Final multivariable models

Variable	Coefficient	95% CI	*p*-value
Final multivariable model for affected adults (*n* = 39) R^2^ 0.42			
Previous OBPI surgery (Reference: No surgery)	0.13	0.01, 0.25	0.040
Employed in manual work (Reference: Not working)	0.02	−0.14, 0.17	0.835
Employed in non-manual work (Reference: Not working)	0.18	0.06, 0.30	0.005
Receives disability benefits related to OBPI (Reference: No benefits)	−0.18	−0.31, − 0.06	0.005
Has partner (Reference: No partner)	0.14	0.04, 0.25	0.009
Final multivariable model for parents (*n* = 63) R^2^ 0.22			
Previous OBPI surgery for child (Reference: No surgery)	−0.0050	− 0.06, 0.05	0.869
Working (parent) (Reference: Not working)	0.11	0.02, 0.20	0.024
Has ≥1 medical condition (parent) (Reference: No medical conditions)	−0.10	− 0.16, − 0.04	0.001

As before, previous OBPI surgery for the child was not associated with the parent utility score in the univariable regression model (95% CI (− 0.14, 0.02), *p* = 0.116). Details of the parent/child univariable regression analyses can be found in Additional files 3 and 4. Previous OBPI surgery for the child, parental employment status and having one or more medical condition (for the parent) were included in the final multivariable model for the parents. The backward elimination steps used to determine this final model are presented in Additional file 5. Six influential observations were removed from the final model. Previous OBPI surgery for the child was not associated with the parent utility score (95% CI (− 0.06, 0.05), *p* = 0.869). Parents who worked appeared to have better utility scores compared to those without employment, after adjusting for the other variables in the final model (95% CI (0.02, 0.20), *p* = 0.024). In contrast, parents with one or more medical condition had worse utility scores than those who reported no health problems, after accounting for the other variables (95% CI (− 0.16, − 0.04), *p* = 0.001).

### Additional analyses (Additional file 6)

The influential observations were re-instated and the final multivariable models were rerun. Interestingly, previous OBPI surgery was no longer associated with the affected adult utility score after the inclusion of the three influential observations (95% CI (− 0.07, 0.22), *p* = 0.281). The inferences for the other variables in the final affected adult multivariable model remained the same. In the final parent multivariable model including influential observations (*n* = 6), parental working status was no longer associated with the parent utility score (95% CI (− 0.10, 0.31), *p* = 0.320) but the inferences for the other variables were unchanged. The presence of missing data from 4 (8%) adult participants and 1 (1%) parent did not impact on the conclusions drawn from the final multivariable models.

## Discussion

Both adults with OBPIs and parents of children with permanent OBPIs had worse utility scores, and therefore quality of life, compared to the general English population. The mean utility score for affected adults was 0.56 (SD 0.28) and the mean utility score for the parents was 0.80 (SD 0.19). We also identified characteristics to consider when dealing with utility scores in populations of adults with OBPIs or parents of children with permanent OBPIs. For affected adults: previous OBPI surgery, employment status, disability benefits status and marital status appeared to be associated with the utility score. For parents: employment status and the presence of one or more parental medical condition appeared to be associated with the utility score.

Only one study has previously presented utility scores associated with OBPIs [11]. Culligan et al. assigned utility scores to different combinations of maternal-neonatal outcomes, some of which included permanent OBPIs [11]. A utility score of 0.60 was assigned by this group of clinicians to mild-moderate OBPIs, and it is similar to the affected adult utility score calculated in this study (7% difference). However, the utility score derived by this study is a more reliable and accurate measure of the quality of life associated with OBPIs. Firstly, the utility score was determined using a validated generic health instrument and involved adults with the injury. Secondly, the affected adult utility score in this study has a clear perspective. As the utility scores assigned by Culligan et al. are for combinations of maternal-neonatal outcomes, it is unclear whether the quality of life was being assessed from the viewpoint of the mother, the baby, or both [11].

The EQ-5D-5 L questionnaire was an appropriate instrument to use to calculate the utility scores in these cohorts. There are currently no OBPI-specific instruments and despite being a generic questionnaire, the domains covered by the EQ-5D-5 L, particularly self-care, usual activities, pain/discomfort and anxiety/depression were relevant to OBPIs. The best way of measuring and integrating the ‘spillover’ effects (the impact on caregivers and family members) of healthcare interventions into cost-effectiveness analyses is still debated [25] and generic health status instruments may not fully capture the ‘spillover’ effects, particularly the potential positive aspects of caring [25]. Despite this, the EQ-5D questionnaire has been able to detect changes in health-related quality of life in caregivers of children with major congenital anomalies [26] and it appears to be useful in measuring family members’ quality of life in economic evaluations [27]. The EQ-5D-5 L questionnaire is also the preferred health status instrument for NICE [28]. This means that the quality adjusted life years (QALYs) calculated from these utility scores would be comparable with the QALYs in other health technology assessments reviewed by NICE.

We mapped the EQ-5D-5 L responses to an EQ-5D-3 L valuation set as recommended by NICE using the *eq5dmap* command in Stata [19]. The command is a mapping process based on a system of ordinal regressions and does not assume that the domains of the EQ-5D instrument are statistically independent [19, 29]. We used the EuroQoL Group dataset [30, 31] as the reference for the mapping process. This dataset consists of a younger population and covers a broader range of diseases compared to the alternative reference dataset available (National Data Bank for Rheumatic Diseases) [32]. Furthermore, the EuroQoL Group data was generated from 6 European countries, including England, making it the more relevant reference dataset to use [30, 31]. The *eq5dmap* command also adjusts for age and gender [19].

The inability to stratify utility scores by the severity of the condition is a limitation of this study. Only a small proportion of affected adults (28%) and parents (41%) knew the Narakas classification of the injuries, even though it is widely used clinically [33]. As a result, a crude proxy variable was needed and previous OBPI surgery was a reasonable choice. There is some evidence that having lower Narakas scores (I-II) reduces the likelihood of permanent OBPIs, and hence the need for surgery [34]. Similarly, the presence of Horner’s syndrome, indicating Narakas score IV, increases the likelihood of permanent OBPIs and therefore operative management [34]. Previous OBPI surgery was associated with the affected adult utility score but not with the parent utility score in this study. In comparison, one previous study has demonstrated that OBPI surgery had an impact on the child’s family [12].

We did not attempt to measure the quality of life utility scores of the children with OBPIs. Young children may not have the cognitive capacity to comprehend or answer health related questions, and there are few preference-based, health status instruments derived from child populations [35, 36]. A small study (*n* = 18) has assessed the quality of life of adolescents (aged 10 to 17 years old) with permanent OBPIs using the Child Health Questionnaire and the Paediatric Outcomes Data Collection Instrument [37]. Whilst the questionnaire and instrument quantify health-related quality of life, the scores cannot be used to calculate QALYs as they are not preference-based. There is a youth version of the EQ-5D questionnaire (EQ-5D-Y) but there are no valuation sets for this at present. Using the EQ-5D adult values for EQ-5D-Y would misrepresent a child’s health status [38]. As a result, the EQ-5D-Y cannot generate reliable child QALYs for use in economic evaluations.

OBPIs are rare and as such, participants were difficult to recruit. Collaborating with the Erb’s Palsy Group allowed access to their membership, the majority of which would have been eligible to participate in the study. Despite attempts to maximise publicity and recruitment, the response rates were low. One reason for this may be because of the perceived lack of benefit in taking part in the study. The small sample sizes of affected adults and parents of children with permanent OBPIs limited the extent of our statistical investigations. As a result, our study had limited statistical power and we could not confidently detect some true associations. Moreover, our analyses were sensitive to the changes in the sample sizes (see additional analyses) and some associations were no longer evident in those additional analyses conducted on marginally larger samples. For example, previous OBPI surgery went from a positive association with the affected adult utility score to no association. This clinical factor was chosen as a proxy for the severity of OBPIs, but it could be possible that surgery improves function to the extent that it results in better quality of life. Further analyses with larger samples are needed to check whether there are indeed any associations between the factors identified in this study and the utility score in these cohorts. Interestingly, mothers of children with permanent OBPIs had a lower mean utility score (0.79) compared to fathers (0.89). One possible explanation for this could be that mothers might have a greater share of the caring responsibility. It is worth noting, however, that this study had a very small cohort of fathers (8% of the total sample) and an even smaller number of single fathers (1% of the total sample) to test this theory. Further research is needed to investigate whether there is a true difference in the quality of life between mothers and fathers of children with permanent OBPIs.

Due to the cross-sectional nature of the study, there may have been selection biases so the samples in this study may not be fully representative of all adults in the UK with OBPIs or parents of children with permanent OBPIs. Furthermore, as this study was based on questionnaires, the responses may also have been affected by recall biases.

Despite the small sample sizes, this is the first study to directly and robustly assess the quality of life of both adults with OBPIs and parents of children with permanent OBPIs using a validated health-status instrument. The findings from this study represent the best available evidence and provide tailored utility scores that could now be used as part of cost-utility analyses of interventions related to improving the care, health and wellbeing of adults affected by OBPIs and parents of children with permanent OBPIs.

## Conclusions

Adults with OBPIs and parents of children with permanent OBPIs reported worse utility scores, and therefore quality of life, compared to the English general population. Previous OBPI surgery, employment status, disability benefits status and marital status appeared to influence the affected adult utility scores. Parental employment status and the presence of one or more parental medical condition appeared to have an association with the parent utility scores. The utility scores in this study can be used in future economic evaluations related to OBPIs.

## Additional files

Additional file 1: Affected adult univariable regression analyses. Table of affected adult univariable regression analyses. (DOCX 24 kb)

Additional file 2: Backward elimination steps used to determine final multivariable model for affected adults. Table showing backward elimination steps used to determine final multivariable model for affected adults. (DOCX 15 kb)

Additional file 3: Parent univariable regression analyses. Table of parent univariable regression analyses. (DOCX 18 kb)

Additional file 4: Child univariable regression analyses. Table of child univariable regression analyses. (DOCX 18 kb)

Additional file 5: Backward elimination steps used to determine final multivariable model for parents. Table showing backward elimination steps used to determine final multivariable model for parents. (DOCX 16 kb)

Additional file 6: Additional analyses. Final multivariable models with influential observations and analysis investigating impact of missing data. (DOCX 16 kb)

## Abbreviations

NICE: National Institute for Health and Care Excellence
OBPI: Obstetric brachial plexus injury
QALY: Quality adjusted life year
