# Supplementary material for: Obstetric brachial plexus injuries (OBPIs): health-related quality of life in affected adults and parents
Source: Health Qual Life Outcomes. 2018 Nov 15;16:212. doi: 10.1186/s12955-018-1039-z (PMC6238314; doi:10.1186/s12955-018-1039-z)
Supplement: Supplementary file 1 — Affected adult univariable regression analyses. Table of affected adult univariable regression analyses. (DOCX 24 kb) [file 12955_2018_1039_MOESM1_ESM.docx]

| **Affected adult characteristics** | **n (%)** | **Coefficient** | **95% CI** | **R^2^** | **p-value** | |
| --- | --- | --- | --- | --- | --- | --- |
| **Age (years)** | 42 (100) | 0.0005 | -0.0037, 0.0048 | 0.0015 | 0.801 | |
| **Age subcategories (years) Ref ≤24** |  |  |  |  | 0.875 | |
| 25-39 | 42 (100) | 0.03 | -0.11, 0.17 | 0.03 | 0.656 | |
| 40-54 |  | -0.09 | -0.46, 0.28 |  | 0.610 | |
| ≥55 |  | 0.04 | -0.15, 0.24 |  | 0.649 | |
| **Gender Ref Male** | 42 (100) | -0.06 | -0.21, 0.09 | 0.01 | 0.445 | |
| Female |  |  |  |  |  |  |
| **Marital status Ref No partner** | 42 (100) | 0.14 | -0.02, 0.29 | 0.07 | 0.077 | |
| Partner |  |  |  |  |  |  |
| **Education status Ref secondary school or college** | 42 (100) | -0.0046 | -0.17, 0.16 | 0.0001 | 0.954 | |
| Higher education |  |  |  |  |  |  |
| **Employment status Ref Not working** |  |  |  |  | 0.284 | |
| Manual work | 42 (100) | 0.06 | -0.14, 0.27 | 0.06 | 0.525 | |
| Non-manual work |  | 0.13 | -0.03, 0.29 |  | 0.118 | |
| **Disability benefits No benefits** | 42 (100) | -0.26 | -0.42, -0.10 | 0.26 | 0.002 | |
| Receives benefits related to OBPI |  |  |  |  |  |  |
| **Handedness Ref Left** | 42 (100) | 0.02 | -0.13, 0.17 | 0.0021 | 0.758 | |
| Right |  |  |  |  |  |  |
| **Injury site Ref Left** |  |  |  |  |  | |
| Right | 42 (100) | -0.11 | -0.26, 0.05 | 0.14 | 0.174 | |
| Both |  | Omitted as only one observation | | | |  |
| **Narakas Ref Know Narakas** | 42 (100) | -0.02 | -0.26, 0.22 | 0.0010 | 0.876 | |
| Don’t know Narakas |  |  |  |  |  |  |
| **Previous OBPI surgery Ref No** | 42 (100) | -0.06 | -0.21, 0.09 | 0.01 | 0.411 | |
| Yes |  |  |  |  |  |  |
| **Has ≥ 1 medical condition**  **Ref None** | 42 (100) | -0.15 | -0.29, -0.02 | 0.09 | 0.028 | |
| Yes |  |  |  |  |  |  |
| **Has ≥ 1 cardiovascular condition Ref None** | 42 (100) | -0.07 | -0.32, 0.17 | 0.01 | 0.548 | |
| Yes |  |  |  |  |  |  |
| **Has ≥ 1 respiratory condition**  **Ref None** | 42 (100) | 0.13 | -0.04, 0.30 | 0.03 | 0.126 | |
| Yes |  |  |  |  |  |  |
| **Has ≥ 1 gastrointestinal condition Ref None** | 42 (100) | -0.13 | -0.27, 0.02 | 0.01 | 0.087 | |
| Yes |  |  |  |  |  |  |
| **Has ≥ 1 musculoskeletal condition Ref None** | 42 (100) | -0.28 | -0.46, -0.10 | 0.26 | 0.003 | |
| Yes |  |  |  |  |  |  |
| **Has ≥ 1 neurological condition**  **Ref None** | 42 (100) | -0.17 | -0.45, 0.10 | 0.03 | 0.210 | |
| Yes |  |  |  |  |  |  |
| **Has ≥ 1 endocrine condition**  **Ref None** | 42 (100) | -0.13 | -0.36, 0.10 | 0.02 | 0.270 | |
| Yes |  |  |  |  |  |  |
| **Has ≥ 1 mental health condition**  **Ref None** | 42 (100) | -0.15 | -0.43, 0.13 | 0.03 | 0.282 | |
| Yes |  |  |  |  |  |  |
| **Has ≥ 1 other condition**  **Ref None** | 42 (100) | -0.01 | -0.13, 0.11 | 0.0002 | 0.876 | |
| Yes |  |  |  |  |  |  |
|  |  |  |  |  |  | |
|  | | | | | |  |

**Affected adult univariable regression analyses**
