# Supplementary material for: Obstetric brachial plexus injuries (OBPIs): health-related quality of life in affected adults and parents
Source: Health Qual Life Outcomes. 2018 Nov 15;16:212. doi: 10.1186/s12955-018-1039-z (PMC6238314; doi:10.1186/s12955-018-1039-z)
Supplement: Supplementary file 2 — Backward elimination steps used to determine final multivariable model for affected adults. Table showing backward elimination steps used to determine final multivariable model for affected adults. (DOCX 15 kb) [file 12955_2018_1039_MOESM2_ESM.docx]

| **Full model** | **Variables of full model** | **Test model** | **Variables of test model** | **Tested variable** | **Wald test**  **(p-value)** |
| --- | --- | --- | --- | --- | --- |
| M_1_ | U=I+Su+Em+Db+Med+M+A+G+Ed | M_2_ | U=I+Su+Em+Db+Med+M+A+G | Ed | 1.000 |
| M_2_ | U=I+Su+Em+Db+Med+M+A+G | M_3_ | U=I+Su+Em+Db+M+A+G | Med | 0.985 |
| M_3_ | U=I+Su+Em+Db+M+A+G | M_4_ | U=I+Su+Em+Db+M+G | A | 0.361* |
| M_3_ | U=I+Su+Em+Db+M+A+G | M_5_ | U=I+Su+Em+Db+A+G | M | 0.209* |
| M_3_ | U=I+Su+Em+Db+M+A+G | M_6_ | U=I+Su+Em+Db+M+A | G | 0.189 |
| M_6_ | U=I+Su+Em+Db+M+A | M_7_ | U=I+Su+Em+Db+M | A | 0.477 |
| M_7_ | U=I+Su+Em+Db+M | M_8_ | U=I+Su+Db+M | Em | 0.061* |
| M_7_ | U=I+Su+Em+Db+M | M_9_ | U=I+Su+Em+Db | M | 0.049 |
| M_7_ | U=I+Su+Em+Db+M | M_10_ | U=I+Su+Em+M | Db | 0.001 |
|  |  |  |  |  |  |
| * variable retained as influencing effect size of other parameters by >30% | | | | | |
| U | Utility score | Med | Has ≥1 medical condition |  |  |
| I | Intercept | M | Marital status |  |  |
| Su | Previous OBPI surgery | A | Age |  |  |
| Em | Employment status | G | Gender |  |  |
| Db | Disability benefits status | Ed | Education status |  |  |

**Backward elimination steps used to determine final multivariable model for affected adults**
