# Supplementary material for: Obstetric brachial plexus injuries (OBPIs): health-related quality of life in affected adults and parents
Source: Health Qual Life Outcomes. 2018 Nov 15;16:212. doi: 10.1186/s12955-018-1039-z (PMC6238314; doi:10.1186/s12955-018-1039-z)
Supplement: Supplementary file 3 — Parent univariable regression analyses. Table of parent univariable regression analyses. (DOCX 18 kb) [file 12955_2018_1039_MOESM3_ESM.docx]

| **Parent characteristics** | **n (%)** | **Coefficient** | **95% CI** | **R^2^** | **p-value** |
| --- | --- | --- | --- | --- | --- |
| **Age (years)** | 69 (100) | 0.0005 | -0.0047, 0.0056 | 0.0003 | 0.859 |
| **Age subcategories (years) Ref ≤35** |  |  |  |  | 0.854 |
| 36-45 | 69 (100) | -0.02 | -0.11, 0.07 | 0.0035 | 0.646 |
| ≥46 |  | -0.03 | -0.15, 0.10 |  | 0.663 |
| **Gender Ref Male** | 69 (100) | -0.11 | -0.23, 0.02 | 0.02 | 0.087 |
| Female |  |  |  |  |  |
| **Relationship Ref Father** | 69 (100) | -0.11 | -0.23, 0.02 | 0.02 | 0.087 |
| Mother |  |  |  |  |  |
| **Marital status Ref No partner** | 69 (100) | -0.01 | -0.14, 0.12 | 0.0004 | 0.892 |
| With partner |  |  |  |  |  |
| **Education status Ref secondary school/college** | 69 (100) | 0.01 | -0.08, 0.10 | 0.0006 | 0.832 |
| Higher education |  |  |  |  |  |
| **Employment Ref Not working** | 69 (100) | 0.15 | -0.05, 0.36 | 0.07 | 0.140 |
| Working |  |  |  |  |  |
| **Has ≥ 1 medical condition Ref None** | 69 (100) | -0.14 | -0.22, -0.06 | 0.13 | 0.001 |
| Yes |  |  |  |  |  |
| **Has ≥ 1 cardiovascular condition Ref None** | 69 (100) | -0.09 | -0.14, -0.04 | 0.01 | 0.001 |
| Yes* |  |  |  |  |  |
| **Has ≥ 1 respiratory condition Ref None** | 69 (100) | -0.11 | -0.24, 0.02 | 0.05 | 0.086 |
| Yes |  |  |  |  |  |
| **Has ≥ 1 gastrointestinal condition Ref None** | 69 (100) | 0.06 | -0.11, 0.22 | 0.01 | 0.507 |
| Yes |  |  |  |  |  |
| **Has ≥ 1 musculoskeletal condition Ref None** | 69 (100) | -0.28 | -0.45, -0.12 | 0.30 | 0.001 |
| Yes |  |  |  |  |  |
| **Has ≥ 1 neurological condition Ref None** | 69 (100) | -0.14 | -0.33, 0.05 | 0.08 | 0.142 |
| Yes |  |  |  |  |  |
| **Has ≥ 1 endocrine condition Ref None** | 69 (100) | -0.14 | -0.31, 0.03 | 0.08 | 0.099 |
| Yes |  |  |  |  |  |
| **Has ≥ 1 mental health condition Ref None** | 69 (100) | -0.23 | -0.40, -0.07 | 0.16 | 0.007 |
| Yes** |  |  |  |  |  |
| **Has ≥ 1 oncological condition Ref None** | 69 (100) | -0.30 | -0.63, 0.03 | 0.10 | 0.072 |
| Yes* |  |  |  |  |  |
| **Has ≥ 1 other condition Ref None** | 69 (100) | -0.01 | -0.17, 0.14 | 0.0004 | 0.869 |
| Yes |  |  |  |  |  |
|  |  |  |  |  |  |
| * Only 3 observations ** Only 8 observations | | | | | |

**Parent univariable regression analyses**
