# Supplementary material for: Obstetric brachial plexus injuries (OBPIs): health-related quality of life in affected adults and parents
Source: Health Qual Life Outcomes. 2018 Nov 15;16:212. doi: 10.1186/s12955-018-1039-z (PMC6238314; doi:10.1186/s12955-018-1039-z)
Supplement: Supplementary file 4 — Child univariable regression analyses. Table of child univariable regression analyses. (DOCX 18 kb) [file 12955_2018_1039_MOESM4_ESM.docx]

| **Child characteristics** | **n (%)** | **Coefficient** | **95% CI** | **R^2^** | **p-value** |
| --- | --- | --- | --- | --- | --- |
| **Age (years)** | 69 (100) | -0.0012 | -0.01, 0.01 | 0.0013 | 0.748 |
| **Age subcategories (years) Ref 1** |  |  |  |  | 0.7257 |
| 2-5 | 69 (100) | 0.0024 | -0.10, 0.10 | 0.02 | 0.963 |
| 6-12 |  | -0.07 | -0.20, 0.06 |  | 0.313 |
| 13-17 |  | -0.01 | -0.13, 0.11 |  | 0.857 |
| **Gender Ref Male** | 69 (100) | -0.01 | -0.10, 0.09 | 0.0002 | 0.906 |
| Female |  |  |  |  |  |
| **Education status Ref Too young for school** |  |  |  |  | 0.829 |
| Primary school | 69 (100) | 0.02 | -0.08, 0.12 | 0.01 | 0.687 |
| Secondary school/college |  | -0.02 | -0.12, 0.09 |  | 0.760 |
| **Previous OBPI surgery Ref No** | 69 (100) | -0.06 | -0.14, 0.02 | 0.03 | 0.116 |
| Yes |  |  |  |  |  |
| **Narakas Ref Knowing** | 69 (100) | 0.02 | -0.07, 0.12 | 0.0042 | 0.602 |
| Not knowing Narakas |  |  |  |  |  |
| **Have ≥ 1 medical condition Ref None** | 69 (100) | -0.03 | -0.12, 0.07 | 0.0047 | 0.587 |
| Yes |  |  |  |  |  |
| **Has ≥ 1 cardiovascular condition Ref None** | 69 (100) | 0.02 | -0.08, 0.13 | 0.0010 | 0.661 |
| Yes |  |  |  |  |  |
| **Has ≥ 1 respiratory condition Ref None** | 69 (100) | -0.09 | -0.26, 0.09 | 0.03 | 0.336 |
| Yes |  |  |  |  |  |
| **Has ≥ 1 gastrointestinal condition Ref None** | 69 (100) | 0.10 | -0.06, 0.26 | 0.01 | 0.206 |
| Yes |  |  |  |  |  |
| **Has ≥ 1 musculoskeletal condition Ref None** | 69 (100) | 0.20 | 0.15, 0.24 | 0.03 | <0.001 |
| Yes* |  |  |  |  |  |
| **Has ≥ 1 neurological condition Ref None** | 69 (100) | 0.04 | -0.06, 0.13 | 0.0037 | 0.431 |
| Yes |  |  |  |  |  |
| **Has ≥ 1 endocrine condition Ref None** | 69 (100) | Omitted as no observations | | | |
| Yes |  |  |  |  |  |
| **Has ≥ 1 mental health condition Ref None** | 69 (100) | -0.12 | -0.28, 0.03 | 0.04 | 0.120 |
| Yes |  |  |  |  |  |
| **Has ≥ 1 oncology condition Ref None** | 69 (100) | Omitted as no observations | | | |
| Yes |  |  |  |  |  |
| **Has ≥ 1 other condition Ref None** | 69 (100) | 0.05 | -0.05, 0.14 | 0.01 | 0.336 |
| Yes |  |  |  |  |  |
|  |  |  |  |  |  |
| * Only 2 observations | | | | | |

**Child univariable regression analyses**
