# Supplementary material for: Obstetric brachial plexus injuries (OBPIs): health-related quality of life in affected adults and parents
Source: Health Qual Life Outcomes. 2018 Nov 15;16:212. doi: 10.1186/s12955-018-1039-z (PMC6238314; doi:10.1186/s12955-018-1039-z)
Supplement: Supplementary file 5 — Backward elimination steps used to determine final multivariable model for parents. Table showing backward elimination steps used to determine final multivariable model for parents. (DOCX 16 kb) [file 12955_2018_1039_MOESM5_ESM.docx]

| **Full model** | **Variables of full model** | **Test model** | **Variables of test model** | **Tested variable** | **Wald test**  **(p-value)** |
| --- | --- | --- | --- | --- | --- |
| N_1_ | U=I+Su+A+G+M+Em+  Ed+Med+CA+CMed | N_2_ | U=I+Su+A+G+Em+  Ed+Med+CA+CMed | M | 0.967 |
| N_2_ | U=I+Su+A+G+Em+  Ed+Med+CA+CMed | N_3_ | U=I+Su+A+G+Em+  Ed+Med+CMed | CA | 0.933 |
| N_3_ | U=I+Su+A+G+Em+  Ed+Med+CMed | N_4_ | U=I+Su+A+G+Em+  Med+CMed | Ed | 0.759 |
| N_4_ | U=I+Su+A+G+Em+  Med+CMed | N_5_ | U=I+Su+G+Em+  Med+CMed | A | 0.735* |
| N_4_ | U=I+Su+A+G+Em+  Med+CMed | N_6_ | U=I+Su+A+G+Em+  Med | CMed | 0.682 |
| N_6_ | U=I+Su+A+G+Em+Med | N_7_ | U=I+Su+G+Em+Med | A | 0.717 |
| N_7_ | U=I+Su+G+Em+Med | N_8_ | U=I+Su+Em+Med | G | 0.118 |
| N_8_ | U=I+Su+Em+Med | N_9_ | U=I+Su+Med | Em | 0.104* |
| N_8_ | U=I+Su+Em+Med | N_10_ | U=I+Su+Em | Med | 0.007 |
|  | |  |  |  |  |
| * variable retained as influencing effect size of other parameters by >30% | | | | | |
| U | Utility score | G | Gender (parent) | Med | Parent has ≥1 medical condition |
| I | Intercept | M | Marital status (parent) | CA | Child age (continuous) |
| Su | Previous OBPI surgery (child) | Ed | Education status (parent) | CMed | Child has ≥1 medical condition |
| A | Age (continuous) (parent) | Em | Employment status (parent) |  |  |
|  |  |  |  |  |  |
|  | |  |  |  |  |

**Backward elimination steps used to determine final multivariable model for parents**
