# Supplementary material for: Obstetric brachial plexus injuries (OBPIs): health-related quality of life in affected adults and parents
Source: Health Qual Life Outcomes. 2018 Nov 15;16:212. doi: 10.1186/s12955-018-1039-z (PMC6238314; doi:10.1186/s12955-018-1039-z)
Supplement: Supplementary file 6 — Additional analyses. Final multivariable models with influential observations and analysis investigating impact of missing data. (DOCX 16 kb) [file 12955_2018_1039_MOESM6_ESM.docx]

| **Final multivariable model for affected adults with influential observations (n=42) R^2^ 0.37** | | | | |
| --- | --- | --- | --- | --- |
| **Variable** | **Coefficient** | **95% CI** | **p-value** |  |
| Previous OBPI surgery | 0.08 | -0.07, 0.22 | 0.281 |  |
| Employed in manual work | 0.07 | -0.10, 0.24 | 0.412 |  |
| Employed in non-manual work | 0.19 | 0.02, 0.35 | 0.031 |  |
| Receives disability benefits related to OBPI | -0.23 | -0.39, -0.07 | 0.005 |  |
| Has partner | 0.14 | 0.01, 0.27 | 0.039 |  |
|  | | | | |
| **Final multivariable model for parents with influential observations (n=69) R^2^ 0.18** | | | | |
| **Variable** | **Coefficient** | **95% CI** | **p-value** |  |
| Previous OBPI surgery for child | -0.03 | -0.10, 0.03 | 0.314 |  |
| Working (parent) | 0.10 | -0.10, 0.31 | 0.320 |  |
| Has ≥1 medical condition (parent) | -0.12 | -0.19, -0.05 | 0.001 |  |

**Additional analyses**

| **Comparison of mean utility scores in participants with missing data and participants with complete data for variables in final multivariable model for affected adults** | | | | | |
| --- | --- | --- | --- | --- | --- |
|  | **Missing data mean (n=4)** | **Complete data mean (n=44)** | **Difference** | **95% CI** | **p- value** |
| Utility score | 0.48 | 0.57 | -0.09 | -0.47, 0.29 | 0.542 |
|  | | | | | |

There were 4 participants with missing data for the variables included in the final multivariable model for the affected adults. Welch’s two-sample t-test was used to compare the mean utility scores of these 4 participants with those that had complete data for these variables. There was no difference between the mean utility scores (95% CI (-0.47, 0.29), p=0.542). This meant that the participants with missing data would not have affected the inferences drawn from the final multivariable model for affected adults. There was only 1 participant with missing data for the variables in the final multivariable model for the parents. This participant would not have affected the inferences drawn from the final multivariable model for the parents.
